# Indoleamine 2,3‐dioxygenase 1 inhibition reverses cancer‐associated fibroblast‐mediated immunosuppression in high‐grade serous ovarian cancer

**DOI:** 10.1002/2211-5463.70126

**Published:** 2025-10-13

**Authors:** Hyewon Lee, Jung Yoon Ho, In Sun Hwang, Youn Jin Choi

**Affiliations:** ^1^ Department of Obstetrics and Gynecology, Seoul St. Mary's Hospital, College of Medicine The Catholic University of Korea Seoul Korea; ^2^ Cancer Research Institute, College of Medicine The Catholic University of Korea Seoul Korea

**Keywords:** cancer‐associated fibroblasts, IDO1, immunosuppression, ovarian cancer, tumor microenvironment

## Abstract

Cancer‐associated fibroblasts (CAFs) contribute to immunosuppression in the ovarian cancer microenvironment, partly through upregulation of indoleamine 2,3‐dioxygenase 1 (IDO1). This study examined CAF‐mediated suppression of T‐cell function and the potential of IDO1 inhibition to reverse these effects. CAFs from high‐grade serous ovarian cancer (HGSOC) patients exhibited increased IDO1, COX2, and PD‐L1 expression upon interaction with activated T cells, along with elevated immunosuppressive cytokines. CAFs suppressed T‐cell proliferation and induced PD‐1 expression in CD4+ and CD8+ T cells, effects reversed by epacadostat. IDO1 inhibition enhanced T‐cell proliferation via AKT signaling, restored T‐cell cytotoxicity, and increased ovarian cancer cell apoptosis. These findings suggest that targeting IDO1 may help counteract CAF‐mediated immunosuppression and enhance antitumor immunity in HGSOC.

## Graphical abstract

CAF‐mediated immunosuppression in ovarian cancer is driven by IDO1, reducing T‐cell function. Inhibiting IDO1 restores T‐cell proliferation and cytotoxicity, increases cancer cell apoptosis, and may help overcome CAF‐induced immune suppression in high‐grade serous ovarian cancer. Targeting IDO1 may improve antitumor immunity.

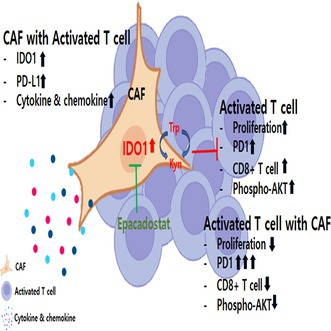

**Abbreviations**
AKT: protein kinase B, a key signaling molecule involved in cell survival and proliferation
ANOVA: analysis of variance
CAF: cancer‐associated fibroblast, a type of fibroblast found in the tumor microenvironment
COX2: cyclooxygenase‐2, an enzyme involved in inflammation
FAP: fibroblast activation protein, a marker for activated fibroblasts
GAPDH: glyceraldehyde‐3‐phosphate dehydrogenase, a housekeeping gene used for normalization in gene expression studies
HGSOC: high‐grade serous ovarian cancer
IDO1: indoleamine 2,3‐dioxygenase 1, an enzyme involved in tryptophan metabolism and immune suppression
IL‐6: interleukin‐6, a cytokine involved in inflammation and immune responses
Kyn: kynurenine, a metabolite produced during tryptophan catabolism and involved in immunosuppression
NF: normal fibroblast, fibroblasts from nontumor tissues
OKT3: anti‐human CD3 antibody
PARP: poly (ADP‐ribose) polymerase
PBMC: peripheral blood mononuclear cell
PD‐1: programmed cell death protein 1, an immune checkpoint molecule
PD‐L1: programmed cell death ligand 1, a ligand for PD‐1
PI3K: phosphatidylinositol 3‐kinase, a key enzyme in the PI3K/AKT signaling pathway
STAT3: signal transducer and activator of transcription 3
TME: tumor microenvironment, the environment surrounding a tumor including various cell types and factors
TNC: tenascin C, a marker for cancer‐associated fibroblasts
α‐SMA: alpha‐smooth muscle actin

As one of the most lethal gynecological malignancies, ovarian cancer has a 5‐year relative survival rate of approximately 29% for advanced stage disease [1]. Despite considerable surgical and chemotherapy advances, prognoses for patients with advanced disease remain poor, thus novel therapeutic approaches are urgently required [2]. From previous preclinical and clinical trials, immunotherapy has become a critical treatment strategy for different cancers in clinical practice [3]; however, its role in ovarian cancer remains limited, with clinical trials showing only modest 10%–15% response rates to immune checkpoint inhibitor monotherapy [4].

As a key factor in cancer progression/treatment resistance, the tumor microenvironment (TME) is comprised of many cell types, including immune, cancer, and stromal cells [5, 6]. Among these cell types, cancer‐associated fibroblasts (CAFs) have garnered significant research attention due to their multifaceted actions in metastasis, tumor growth, and immunosuppression [7, 8]. CAFs are activated fibroblasts which occur in the tumor stroma and express specific markers, such as and fibroblast activation protein (FAP), α‐smooth muscle actin (α‐SMA), and platelet‐derived growth factor receptors (PDGFRs) [9, 10], but they also promote tumor progression via different mechanisms, including growth factor, cytokine, and extracellular matrix protein secretion [10, 11]. One intriguing aspect of CAF biology is their potential to modulate antitumor immune responses [2, 12]; emerging evidence now suggests that CAFs create an immunosuppressive microenvironment by secreting different factors that inhibit T‐cell function and promote immunosuppressive cell recruitment [13, 14, 15]. However, how CAFs exert these immunosuppressive effects in ovarian cancer remain unclear.

T cells have key roles in antitumor immunity, with CD8+ cytotoxic T cells particularly important in direct tumor cell killing [16]. T‐cell activation and function are tightly regulated by different signaling pathways, for example, phosphatidylinositol 3‐kinase/protein kinase B (PI3K/Akt) signaling, which promote T‐cell survival, effector, and proliferation functions [17, 18]. Additionally, immune checkpoint molecules (e.g., programmed cell death protein 1 (PD‐1)) negatively regulate T‐cell responses upon ligand interaction [19].

Indoleamine 2,3‐dioxygenase 1 (IDO1) is a key immunosuppressive enzyme expressed in different cells in the TME, including CAFs [20, 21]. The enzyme catalyzes the rate‐limiting step in tryptophan catabolism, depleting tryptophan and accumulating the immunosuppressive metabolite kynurenine (Kyn) [22]. This can cause T‐cell dysfunction and promote regulatory T‐cell differentiation [21, 23]. Critically, IDO1 inhibitors (e.g., epacadostat) have shown good results in preclinical studies and are currently in clinical trials for various cancers, including melanoma, nonsmall cell lung cancer, bladder cancer, head and neck squamous cell carcinoma, and ovarian cancer [24, 25, 26, 27].

Despite increased CAF recognition as an important player in the HGSOC TME [13, 28], their specific effects on T‐cell function and their potential to target CAF‐mediated immunosuppression remain underexplored. Here, we investigated the immunosuppressive properties of HGSOC‐derived CAFs to specifically examine their impact on T‐cell proliferation, activation, and effector functions. Furthermore, we elucidated IDO1 actions in CAF‐mediated immunosuppression and evaluated IDO1 inhibition as a potential strategy enhancing antitumor T‐cell responses in ovarian cancer.

## Materials and methods

### Study samples

Ovarian tissue samples came from four patients who underwent surgery at St. Mary's Hospital (Seoul, Republic of Korea). Peripheral blood samples were also obtained from two healthy female donors (aged 43 and 44 years). This study was approved by the hospital's Institutional Review Board (Approval number: KC22TISI0006). Written informed consent was provided before participant inclusion. All procedures involving human participants were conducted in accordance with the principles of the Declaration of Helsinki.

### Cell isolation and culture from patients with HGSOC

CAFs were isolated from resected tumor tissues of HGSOC patients. Specimens were washed in phosphate‐buffered saline (PBS; Hyclone, Logan, UT, USA), minced into 1–2 mm^3^ sections, and digested in 500 μg·mL^−1^ liberase TH (Roche, Basel, Switzerland) at 37 °C for 30–60 min. Samples then underwent centrifugation (3 min at 293 ***g*** (RCF)) after which supernatants were discarded. Pellets were resuspended in Dulbecco's modified Eagle medium (DMEM)/F12 (Gibco, Waltham, MA, USA) plus 10% heat‐inactivated fetal bovine serum (FBS; Gibco) and a 1% antibiotic–antimycotic solution (Gibco). Cell suspensions were added to 100 mm culture dishes (Falcon, Corning, NY, USA) and incubated for 7–14 d (5% CO_2_ at 37 °C), allowing CAFs to migrate from tissue fragments. Fibroblasts were then harvested using trypsin–EDTA (Gibco), with cells filtered through a 100 μm cell strainer (SPL, Pocheon, Gyeonggi‐do, South Korea) to generate single‐cell suspensions. These were transferred to new 100 mm culture dishes for expansion. Primary CAFs were used up to passage 10.

### Cell lines and culture

The human ovarian cancer cell line CAOV3 (RRID: CVCL_0201) was obtained from the Korean Cell Line Bank, Korean Cell Line Research Foundation (Seoul, Korea) and cultured in DMEM Medium supplemented with 10% heat‐inactivated FBS. The BRCA1‐deficient ovarian cancer cell line UWB1.289 (RRID: CVCL_B079), purchased from the American Type Culture Collection (ATCC, Manassas, VA, USA), was used to provide a broader genetic context for CAF marker comparison, representing the BRCA1‐mutated subset of ovarian cancers present in our patient cohort. UWB1.289 cells were maintained in a 1:1 mixture of MEGM Medium (prepared using MEBM basal medium and SingleQuot additives from Lonza, Basel, Switzerland) and RPMI 1640 medium (ATCC modification, Gibco), supplemented with 3% heat‐inactivated FBS. All primary cells and cell lines were cultured at 37 °C in a 5% CO_2_ atmosphere.

### Peripheral blood mononuclear cell (PBMC) isolation

Whole blood from healthy donors was used to isolate PBMCs using a Ficoll‐Paque PLUS density gradient centrifugation process (GE Healthcare Life Sciences, Chicago, IL, USA). Buffy coats (diluted 1:1 in PBS) were layered over Ficoll‐Paque PLUS and centrifuged (30 min at 1084 *g* (RCF) at 20 °C) with no brake.

### CAF–PBMC coculture and proliferation assays

For coculture studies, CAFs were added to DMEM/F12 medium plus 10% heat‐inactivated FBS and incubated overnight. To evaluate T lymphocyte expansion, 1 μm carboxyfluorescein succinimidyl ester (CFSE; Invitrogen, Waltham, MA, USA) in PBS was used to stain PBMCs (37 °C for 15 min). Cells were then washed three times in PBS plus heat‐inactivated FBS (5%). Then, PBMCs were added to culture plates either alone or in combination with CAFs, maintaining a PBMC: CAF ratio = 6:1. Cultures were grown for 5 days under two conditions: unstimulated or supplemented with a T‐cell activator, antihuman CD3 antibody (OKT3; 15 ng·mL^−1^; BioLegend, San Diego, CA, USA). Cells then underwent flow cytometry (Flow‐activated cell sorting (FACS) Canto II system; BD Biosciences, Franklin Lakes, NJ, USA) to determine T‐cell proliferation.

### Flow cytometry

This step was performed using a FACS Canto II system where single‐cell suspensions were labeled with antibodies (Table S1). Briefly, 0.5–2 × 10^6^ cells/sample were incubated in 50 μL FACS buffer (1% bovine serum albumin (Biosesang, Seongnam, Gyeonggi‐do, South Korea)) in PBS with appropriate antibodies for 20 min at 2–8 °C. Cells were then washed in FACS buffer and filtered through a 35 μm cell strainer cap (on FACS tubes, BD Biosciences) to ensure single‐cell suspensions. Data were acquired by the instrument and analyzed in FlowJo software (version 10.9.0, BD Biosciences).

### Intracellular phospho‐AKT‐specific flow cytometry

Cells were fixed/permeabilized (BD Cytofix/Cytoperm™ Fixation/Permeabilization Kit, BD Biosciences) after surface marker staining. The fixation/permeabilization solution was added to cell suspensions and incubated for 20 min at room temperature to fix cells and preserve their phosphorylation status. Next, to remove excess fixative and permeabilize cell membranes, cells were washed in BD Perm/Wash™ buffer which facilitated antibody access to intracellular proteins. Cells were then added to a phycoerythrin (PE)‐conjugated anti‐phospho‐AKT (Ser473) antibody (BioLegend), which specifically bound to phosphorylated AKT. After staining for 30 min in the dark at room temperature, cells were rewashed in BD Perm/Wash™ Buffer to remove unbound antibodies. Finally, once resuspended in FACS buffer, cells were analyzed by flow cytometry to quantify phospho‐AKT levels in individual T cells and provide AKT signaling status in specific T‐cell subsets. To evaluate the effect of PI3K/Akt pathway inhibition on T‐cell proliferation, the cells were treated with the PI3K inhibitor LY294002 (MedChemExpress, Monmouth Junction, NJ, USA) at concentrations of 0, 5, 10, and 20 μm.

### Real‐time polymerase chain reaction (PCR)

Total RNA was extracted from CAFs and cancer cell lines (RNeasy Mini Kit, Qiagen, Hilden, Germany) and RNA concentrations/purity examined using a Nanodrop 2000 spectrophotometer (Thermo Fisher Scientific, Waltham, MA, USA). cDNAs were synthesized from total RNA using the Transcriptor First Strand cDNA Synthesis Kit (Roche, Basel, Switzerland) on a SimpliAmp Thermal Cycler (Applied Biosystems, Waltham, MA, USA), following the manufacturer's instructions. Quantitative real‐time PCR was performed on the ViiA 7 Real‐Time PCR System (Applied Biosystems, Waltham, MA, USA) using SYBR Green PCR Master Mix (Applied Biosystems). Thermal cycling parameters included an initial denaturation at 95 °C for 10 min, followed by 40 cycles of 95 °C for 15 s, 52 °C for 15 s, and 72 °C for 20 s. For accuracy, PCRs were performed three times, with melt curves indicating specificity after PCR. Cycle threshold (Ct) values indicated the cycle number at which fluorescence surpassed the threshold. Relative gene expression was calculated using the 2^−ΔΔCt^ method as described by Livak and Schmittgen (2001) [29]. ΔCt was defined as the difference between the Ct value of the target gene and that of *GAPDH*. The ΔΔCt was calculated by subtracting the mean ΔCt of the calibrator group (CAFs) from the ΔCt of each sample. Fold change values were log2‐transformed for graphical presentation. All experiments were performed in triplicate. Primers used (Table S2) were designed using Primer‐BLAST (NCBI) and synthesized by Cosmo Genetech (Seoul, Korea).

### Cell viability assays

Assays were performed using the EZ‐Cytox analysis kit (Dogen, Seoul, South Korea), which used water‐soluble tetrazolium (WST) salts to measure metabolic cell activity. Briefly, cells were added to 96‐well plates (3 × 10^3^ cells/well) and incubated overnight. Cells were treated with serially diluted concentrations of IDO1 inhibitor (epacadostat) (MedChemExpress, Monmouth Junction, NJ, USA) at 0 μm and at concentrations ranging from 4 μm to 0.0078 μm using a 2‐fold serial dilution and incubated for 5 days. After incubation, EZ‐Cytox reagent was added directly to each well at a volume equivalent to 10% of the total culture volume. Plates were incubated for 1 h at 37 °C to allow the WST reagent react with mitochondrial dehydrogenases in viable cells, generating a water‐soluble formazan dye. Absorbance (450 nm) was measured using a SpectraMax ABS Plus microplate reader (Molecular Devices, San Jose, CA, USA), with a reference wavelength set between 600 nm and 650 nm to account for background interference. Color intensity was directly proportional to living cells. For accuracy and reproducibility, conditions were tested in triplicate.

### Apoptosis assays

CAF and activated T‐cell effects on CAOV3 ovarian cancer cell apoptosis were evaluated using a 6‐well Transwell system (pore size = 0.4 μm, Corning Inc., Corning, NY, USA). Cocultures were established at a 1:1:5 ratio for CAOV3 cells, CAFs, and PBMCs, respectively. CAOV3 cells were added to the lower chambers at 2 × 10^5^ cells/well in DMEM/F12 plus 10% heat‐inactivated FBS. CAFs were seeded in upper inserts at the same density and incubated overnight (37 °C in 5% CO_2_). The next day, PBMCs (pretreated with OKT3 (15 ng·mL^−1^) to activate T cells) were seeded in upper chamber inserts at 1 × 10^6^ cells/insert. Cocultures were maintained for 5 days to facilitate interactions between cells. Subsequently, CAOV3 cell apoptosis was assessed by flow cytometry following cell staining using the FITC Annexin V Apoptosis Detection Kit I (BD Biosciences). Apoptotic cells were quantified to assess CAF and activated T‐cell effects on CAOV3 cell survival.

### Statistical analysis

GraphPad Prism (v.9.0, GraphPad Software Inc., San Diego, CA, USA) was used for statistical analyses. For multiple comparisons, one‐way analysis of variance (ANOVA) followed by Tukey's tests was used. Simple comparisons were analyzed using two‐tailed unpaired t‐tests. A *P* value <0.05 indicated statistical significance. Significant differences were denoted as *P < 0.05, **P < 0.01, ***P < 0.001, and ****P < 0.0001, while ‘ns’ indicated not significant.

## Results

### CAF characterization in HGSOC

**Fig. 1:**
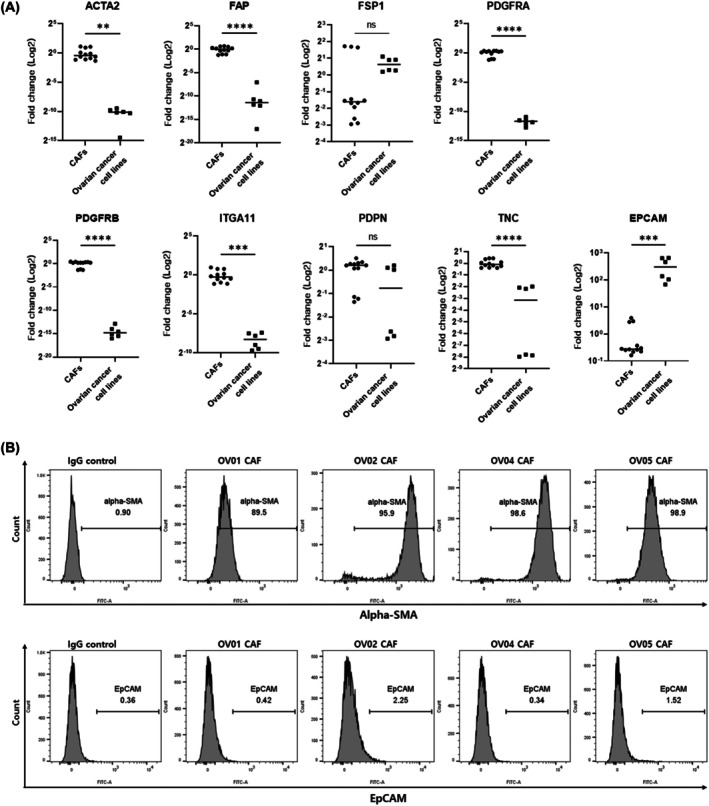
Cancer‐associated fibroblast (CAF) marker characterization in HGSOC. (A) CAF marker mRNA expression in ovarian cancer‐derived CAFs when compared to ovarian cancer cell lines. Real‐time polymerase chain reaction (PCR) was used to evaluate candidate CAF marker levels (*ACTA2*, *FAP*, *FSP1*, *PDGFRA*, *PDGFRB*, *ITGA11*, *PDPN*, and *TNC*) and the epithelial marker *EPCAM* in four HGSOC‐derived CAFs (OV01, OV02, OV04, and OV05) and two ovarian cancer cell lines (UWB1.289 and CAOV3). Expression was normalized to *GAPDH* mRNA levels. Each dot represents an individual replicate across biological replicates (*n* = 4 for CAFs; *n* = 2 for cancer cell lines, each in triplicate). Data are presented as fold change (2^−ΔΔCt^) relative to the mean expression in CAFs. The Y‐axis indicates fold change (log2 scale). Statistical significance was calculated using unpaired t‐tests. **P < 0.01, ***P < 0.001, ****P < 0.0001, and ns = not significant. (B) Flow cytometry analysis of alpha‐SMA and EpCAM expression in primary HGSOC‐derived CAFs from patients (OV01, OV02, OV04, and OV05). Representative histograms and the percentage of positive cells are shown for each CAF sample.

We isolated CAFs from four HGSOC (OV01, OV02, OV04, and OV05), whose clinical details are shown in Table 1. Using real‐time PCR, candidate CAF marker gene mRNA expression levels (ACTA2, FAP, FSP1, PDGFRA, PDGFRB, ITGA11, PDPN, TNC) and the epithelial marker EPCAM were analyzed and compared between CAFs and two ovarian cancer cell lines (UWB1.289 and CAOV3) (Fig. 1A). CAFs demonstrated higher candidate marker levels than ovarian cancer cell lines, except for FSP1 and PDPN. To further characterize primary CAFs, flow cytometry was used to assess the expression of the CAF marker alpha‐SMA and epithelial marker EpCAM (Fig. 1B). All CAFs showed strong alpha‐SMA expression (>89%) and minimal EpCAM expression (<2.3%), confirming their fibroblastic and nonepithelial identity.

**Table 1:** Clinical characteristics of patients with ovarian cancer. BRCA: *Breast Cancer Gene* (BRCA1 and BRCA2); CAF, cancer‐associated fibroblast; WT, *wild‐type* (no mutation detected).

CAF/NF	Age	Type of tumor	Stage	Histological subtype	BRCA status
OV01‐CAF	56	Ovary cancer	IIIC	High‐grade serous carcinoma	*BRCA1* (c.4039_4040del)
OV02‐CAF	63	Ovary cancer	IVB	High‐grade serous carcinoma	*BRCA2* (c.7480C>T)
OV04‐CAF	62	Ovary cancer	IIIC	High‐grade serous carcinoma	WT
OV05‐CAF	66	Ovary cancer	IIIC	High‐grade serous carcinoma	*BRCA1* (c.763G>T)

### Immunosuppressive molecules are upregulated in CAFs after activated T‐cell coculture

**Fig. 2:**
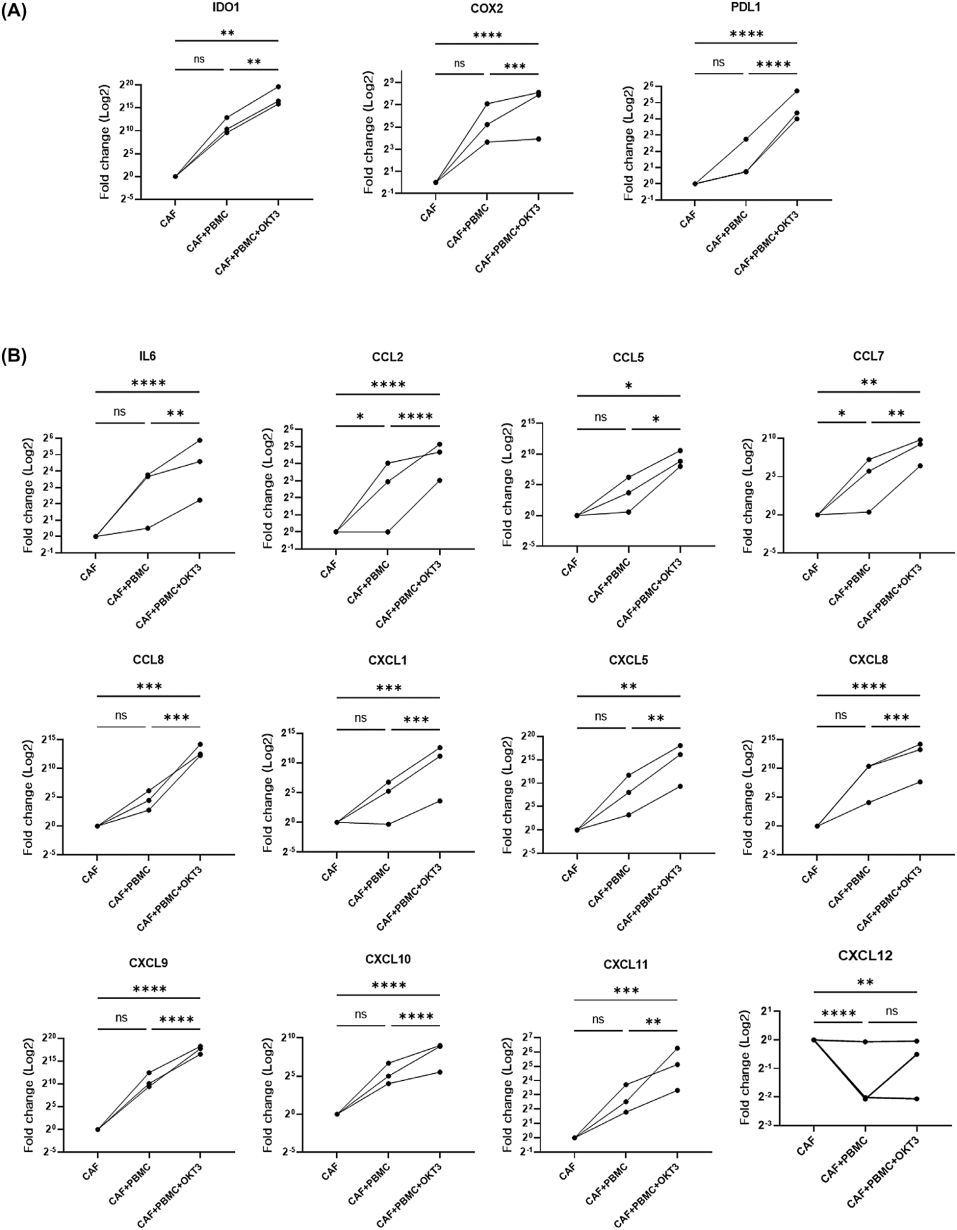
Immunosuppressive molecule, cytokine, and chemokine levels in HGSC CAFs after coculture with peripheral blood mononuclear cells (PBMCs). (A) Real‐time PCR expression analysis of immunosuppressive molecules (*IDO1*, *COX2*, and *PD‐L1*) in CAFs with or without activated PBMC coculture. PBMCs were stimulated with anti‐CD3 antibody (OKT3) (15 ng·mL^−1^) for 5 days. (B) Real‐time PCR expression analysis of cytokine (*IL6*, *CCL2*, *CCL5*, *CCL7*, and *CCL8*) and chemokine (*CXCL1*, *CXCL5*, *CXCL8*, *CXCL9*, *CXCL10*, *CXCL11*, and *CXCL12*) levels in CAFs. Gene expression was calculated using the 2^−ΔΔCt^ method, with CAFs cultured alone as the reference group (CAF = 1; log₂(1) = 0), and data are presented as log2 fold change. Each line represents an independent biological replicate (CAFs from three different patients: OV02, OV04, and OV05). Statistical significance was determined by two‐way ANOVA followed by Tukey's multiple comparison test. (*P < 0.05, **P < 0.01; ***P < 0.001, ****P < 0.0001, and ns = not significant).

We examined immunosuppressive molecule expression, such as *IDO1*, *COX2*, and *PD‐L1*, which are normally expressed in CAFs [30, 31]. To understand their roles in HGSOC CAFs, we investigated mRNA levels (in CAFs) with or without activated PBMCs. *IDO1*, *COX2*, and *PD‐L1* expression was significantly elevated under stimulated conditions in CAFs cocultured with PBMCs when compared with unstimulated conditions (Fig. 2A). Additionally, we examined cytokine (*IL6*, *CCL2*, *CCL5*, *CCL7*, and *CCL8*) and chemokine (*CXCL1*, *CXCL5*, *CXCL8*, *CXCL9*, *CXCL10*, *CXCL11*, and *CXCL12*) levels, which were associated with inflammation and immunosuppression. Most expression levels were increased under stimulated conditions, except for *CXCL12* (Fig. 2B). Thus, coculturing CAFs with activated T cells upregulated immunosuppressive molecules and different cytokines/chemokines, which possibly contributed to an immunosuppressive TME.

### CAFs suppress T‐cell proliferation and promote PD‐1 levels in CD4+ and CD8+ T cells

**Fig. 3:**
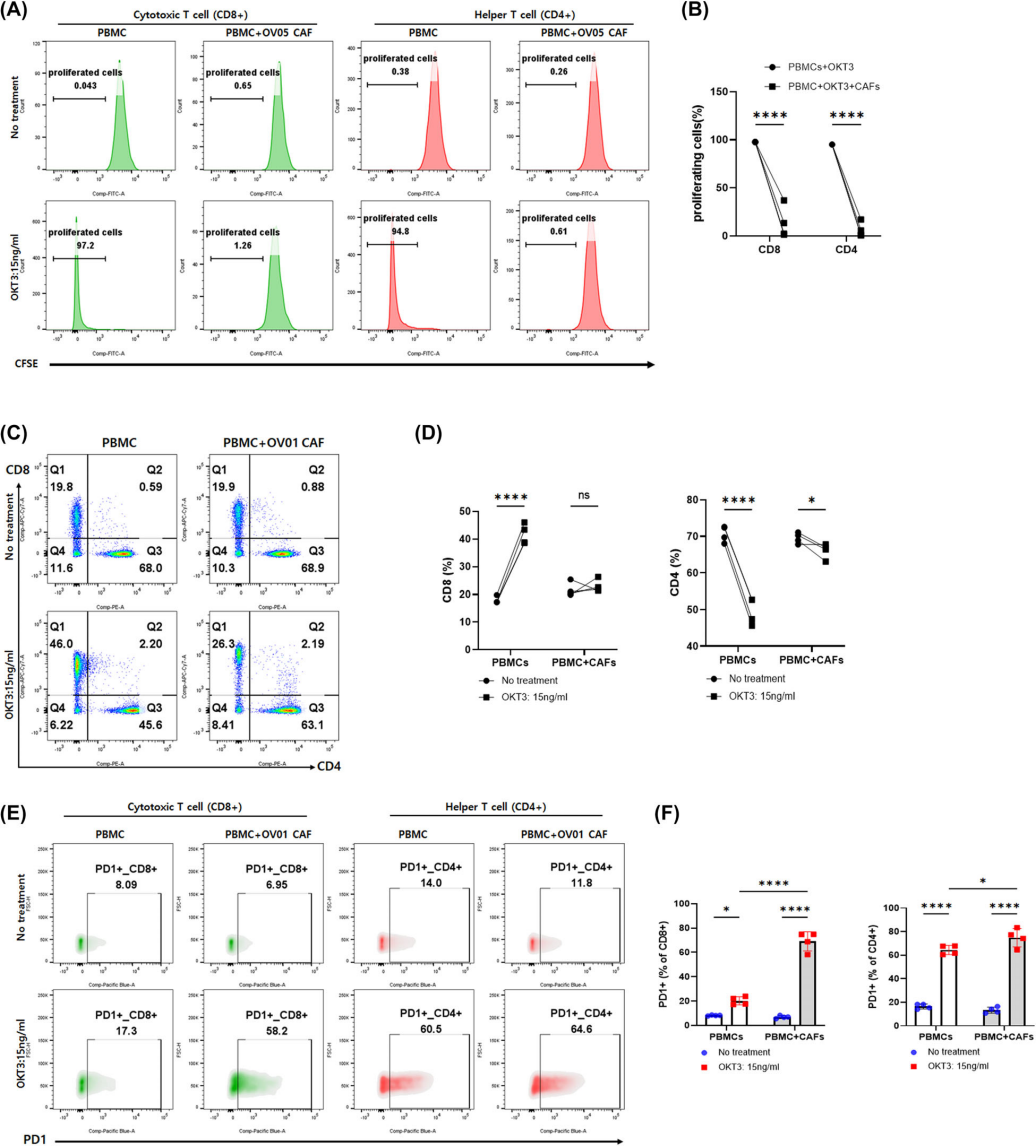
CAF effects on T‐cell proliferation, programmed cell death protein 1 (PD‐1) expression, and CD8 proportions. (A) Representative histograms showing proliferation of CD8+ and CD4+ T cells within PBMCs from healthy donors, as measured by CFSE dilution after 5 days of culture. PBMCs were cultured alone or cocultured with patient‐derived CAFs (OV05) at a PBMC:CAF ratio of 6:1. Cultures were either left unstimulated or stimulated with OKT3 (15 ng·mL^−1^). (B) Quantified CD8+ and CD4+ T‐cell proliferation in PBMCs cultured with or without CAFs under OKT3 stimulation. Data represent mean ± SEM from four independent experiments (*n* = 4, using CAFs from OV01, OV02, OV04, and OV05). (C) Flow cytometry dot plots show CD8+ and CD4+ T‐cell populations in PBMCs cultured alone or cocultured with CAFs (OV01) under OKT3 stimulation for 5 days. (D) Quantified CD8+ and CD4+ T‐cell percentages in PBMCs cultured with or without CAFs under OKT3 stimulation or no treatment (OV01, OV02, OV04, and OV05, *n* = 4). (E) Flow cytometry plots show PD‐1 levels in CD8+ and CD4+ T cells after culture with or without CAFs (OV01) under OKT3 stimulation. (F) Quantified PD‐1 levels in CD8+ and CD4+ T cells in PBMC cultures with or without CAFs under OKT3 stimulation (OV01, OV02, OV04, and OV05, *n* = 4). Statistical significance was determined using unpaired t‐tests; *P < 0.05, ****P < 0.0001, and ns = not significant.

To examine CAF effects on T‐cell proliferation, CFSE‐labeled PBMCs (from healthy donors) were grown for 5 days either alone or with four patient‐derived CAFs (OV01, OV02, OV04, and OV05), in the presence of OKT3 (15 ng·mL^−1^) (Fig. 3). In T cells cultured alone with OKT3, CD4+ and CD8+ T‐cell proliferation increased; however, CAF coculture significantly reduced this proliferation (Fig. 3A, B). Furthermore, post OKT3 stimulation, the CD8+ T‐cell population increased by an average of 23.8% (from 17.9% to 41.7%) without CAFs, while it barely increased by an average of 1.3% (from 21.7% to 23%) in the presence of CAFs (Fig. 3C,D). PD‐1 is a checkpoint protein expressed on T cells that, upon bounding to its ligand, inhibits T‐cell function and suppresses immune responses by downregulating T‐cell inflammatory activity and proliferation [32]. We therefore investigated if CAFs were involved in PD‐1 expression on T cells. During T‐cell activation alone without CAFs, PD‐1 levels increased by an average of 12% (from 8.2% to 20.2%) in CD8+ T‐cell populations and by an average of 48% (from 16.5% to 64.5%) in CD4+ T‐cell populations. In CAF coculture, levels increased by an average of 62.3% (from 6.9% to 69.2%) in CD8+ T‐cell populations and by an average of 61.5% (from 13.4% to 74.9%) in CD4+ T‐cell populations, suggesting that CAFs induced higher PD‐1 levels in CD8+ T cells when compared to CD4+ T cells (Fig. 3E,F). Thus, CAFs not only inhibited CD4+ and CD8+ T‐cell proliferation but also decreased the proportion of CD8+ T cells within the CD3+ T‐cell population and significantly upregulated PD‐1 expression.

### Epacadostat reverses suppressed T‐cell proliferation and CD8+ T‐cell subsets caused by CAFs

**Fig. 4:**
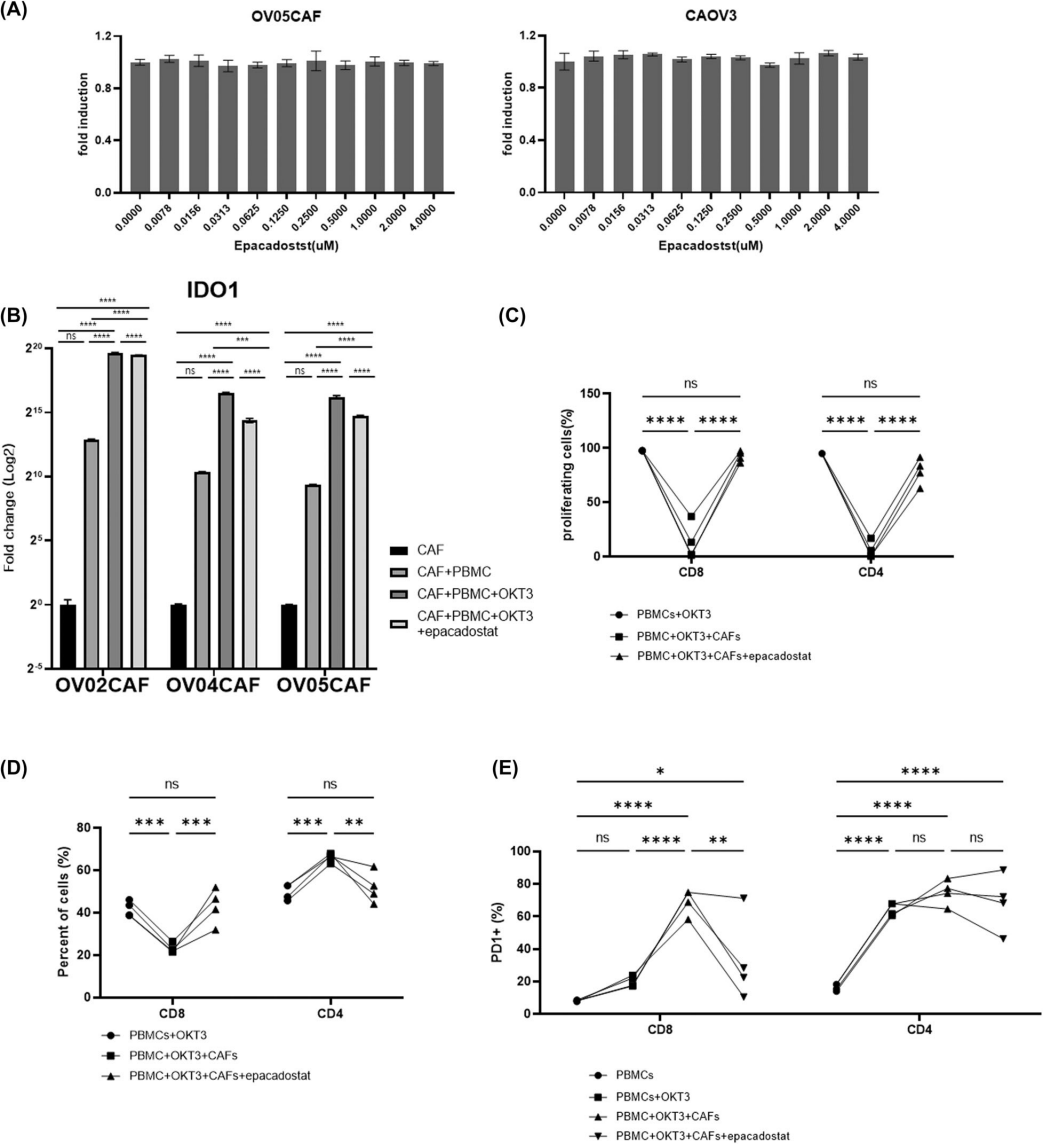
IDO1 inhibition effects by epacadostat on CAF‐induced T‐cell suppression and PD‐1 levels. (A) Epacadostat cytotoxicity toward OV05CAF and CAOV3 cell lines was evaluated using water‐soluble tetrazolium salt (WST) assays. Cells were treated with different epacadostat concentrations (0–4 μm) for 5 days, and cell viability was measured. (B) *IDO1* mRNA expression in CAFs (OV02CAF, OV04CAF, and OV05CAF) was analyzed using real‐time PCR after coculture with activated PBMCs for 5 days, with or without 500 nm epacadostat. (C–E) CFSE‐labeled PBMCs were cocultured with or without CAFs (OV01, OV02, OV04, and OV05) at a PBMC:CAF ratio of 6:1 and cultured for 5 days with OKT3 (15 ng·mL^−1^) stimulation, in the presence or absence of epacadostat (500 nm). (C) Quantified CD4+ and CD8+ T‐cell proliferation (CFSE staining; *n* = 4). (D) Quantified CD8+ and CD4+ T‐cell proportions (*n* = 4). (E) Quantified PD‐1 levels in CD8+ and CD4+ T cells (*n* = 4). Statistical significance was determined using one‐way ANOVA followed by Tukey's multiple comparison tests (*P < 0.05, **P < 0.01, ***P < 0.001, ****P < 0.0001, and ns = not significant).

Epacadostat (IDO1 inhibitor) was used to explore how IDO1 contributed to CAF immunosuppression. Epacadostat cytotoxicity toward OV05CAF (CAF isolated from HGSOC patient OV05) and CAOV3 ovarian cancer cells was measured using WST assays. No significant cytotoxicity was identified when both cell lines were treated with sequential drug concentrations (0–4 μm) (Fig. 4A). In coculture studies with three CAF types, increased IDO1 levels in CAFs after T‐cell coculture were inhibited by 500 nm epacadostat (Fig. 4B). Furthermore, epacadostat restored CD4+ and CD8+ T‐cell proliferation that had been suppressed by CAFs (Fig. 4C) and partially recovered CD8+ cell proportions that had been decreased in CAF coculture (Fig. 4D). Thus, epacadostat appeared to reduce CAF immunosuppressive functions, restore T‐cell proliferation and proportions, and mitigate CAF immunosuppressive effects.

### Epacadostat reduces PD‐1 levels in CD8+ T cells activated by CAFs

We investigated PD‐1 levels in T cells following IDO1 inhibition to determine if CAF‐induced increases in PD‐1 levels in T cells were regulated by IDO1 activity. We treated CAFs and activated T cells with epacadostat and analyzed PD‐1 levels in T cells by flow cytometry. We observed significantly reduced CAF‐induced PD‐1 levels in CD8+ cytotoxic T cells. In contrast, epacadostat effects on PD‐1 levels in CD4+ helper T cells were minimal (Fig. 4E). Thus, CAF‐mediated PD‐1 upregulation in cytotoxic T cells appeared to be regulated via IDO1.

### Epacadostat enhances T‐cell proliferation by restoring phospho‐AKT expression suppressed by CAFs

**Fig. 5:**
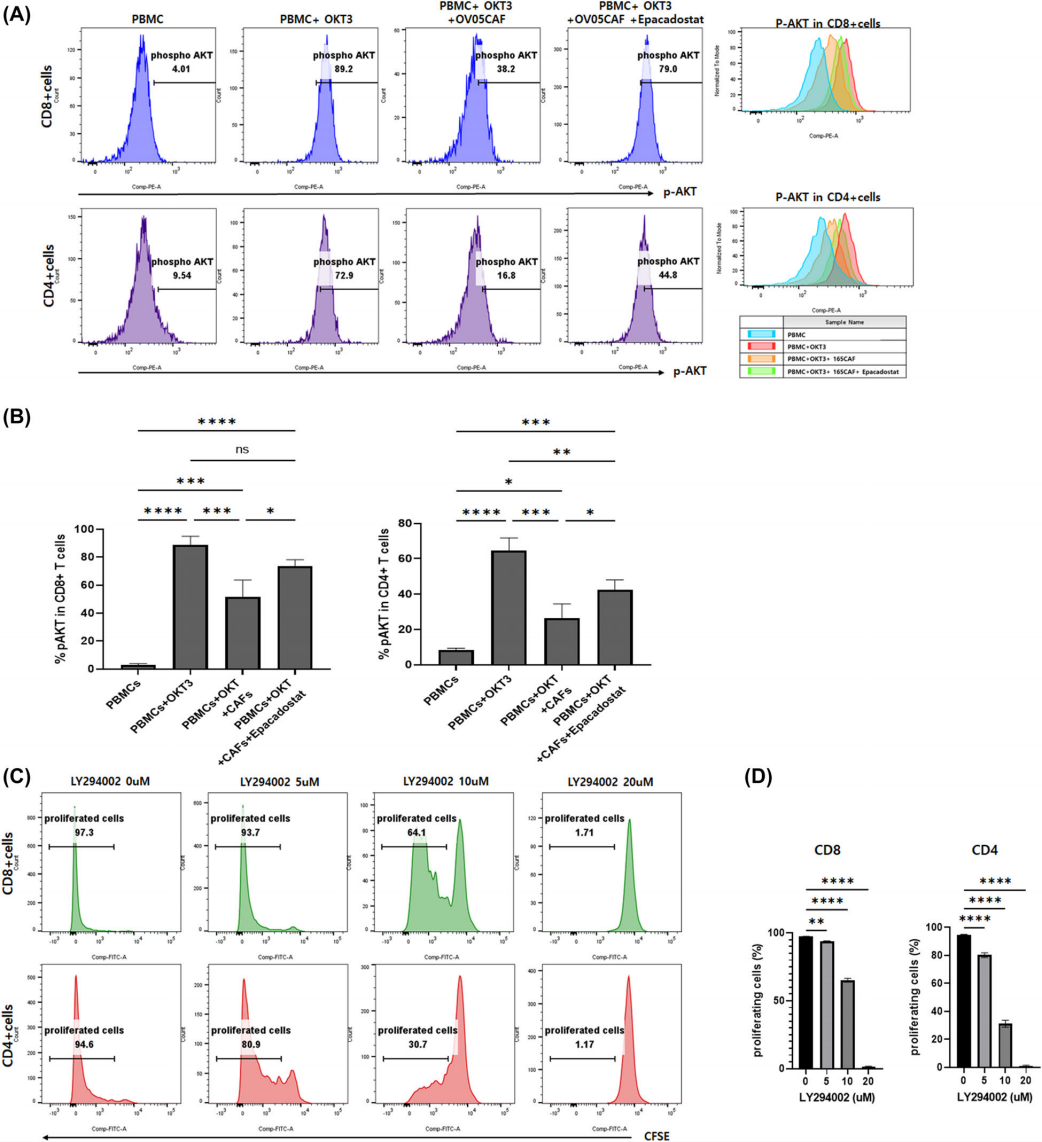
IDO1 inhibition promotes T‐cell proliferation by increasing protein kinase B (Akt) phosphorylation suppressed by CAFs. (A) Flow cytometric analysis of phosphorylated Akt (ser473) in PBMCs. Cells were activated with OKT3 (15 ng·mL^−1^) in the presence or absence of OV05 CAFs at a PBMC:CAF ratio 6:1 and with or without epacadostat (500 nm) for 5 days. Histograms show p‐Akt expression in CD8+ and CD4+ T cells. (B) Quantified p‐Akt levels in CD8+ and CD4+ T cells from experiments using three different CAFs (OV01, OV02, and OV05). (C) Flow cytometry histograms show T‐cell proliferation (CFSE stained) with increasing concentrations of LY294002 (phosphatidylinositol 3‐kinase (PI3K)/Akt pathway inhibitor) (0, 5, 10, and 20 μm). Proliferation was evaluated in CD8+ and CD4+ T cells. (D) Quantified percentages of proliferating CD8+ and CD4+ T cells from (C). Data represent mean ± SEM from three independent experiments (*n* = 3). Statistical significance was determined using one‐way ANOVA followed by Tukey's multiple comparison tests (*P < 0.05, **P < 0.01, ***P < 0.001, ****P < 0.0001, and ns = not significant).

Akt signaling is critical for many T‐cell functions, including proliferation, survival, and differentiation [33, 34]. We examined if suppressed T‐cell proliferation by CAFs was linked to Akt activation and IDO1 expression. Activated PBMCs (from healthy donors) increased Akt phosphorylation in T cells; however, CAF coculture decreased this phosphorylation, suggesting that CAFs affected Akt phosphorylation in T cells. To examine links between IDO1 and T‐cell Akt phosphorylation, we treated cells with epacadostat and observed that IDO1 inhibition increased Akt phosphorylation in T cells (Fig. 5A,B). Additionally, to verify links between Akt phosphorylation and T‐cell proliferation, cells were treated with different concentrations of LY294002 (PI3K/Akt pathway inhibitor, MedChemExpress), which inhibited T‐cell proliferation (Fig. 5C,D). These observations suggested that suppressed T‐cell proliferation by CAFs was possibly mediated via increased IDO1 and decreased Akt phosphorylation levels, indicating a novel T‐cell regulatory mechanism in the TME.

### Epacadostat restores T‐cell cytotoxicity suppressed by CAFs

**Fig. 6:**
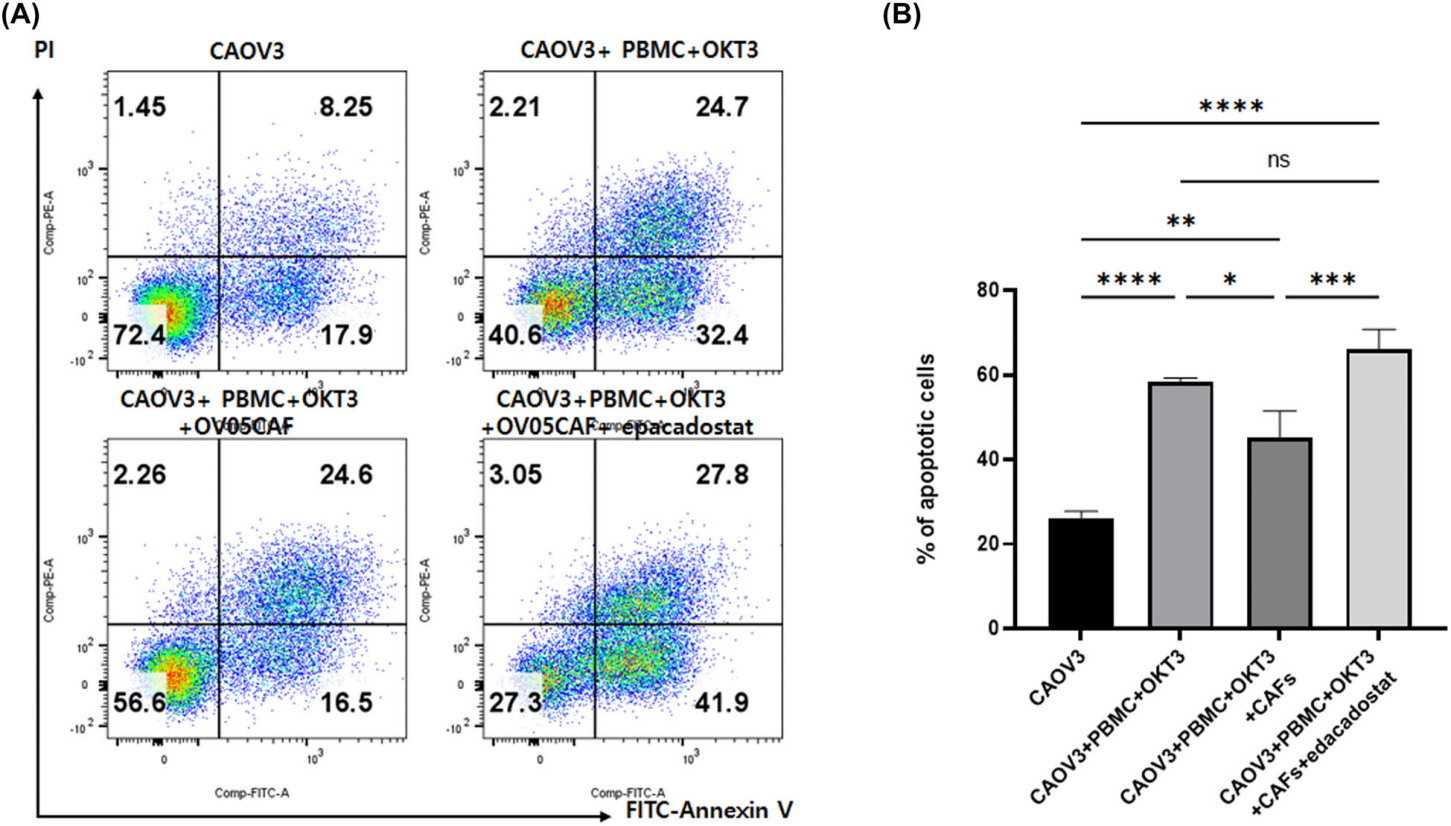
IDO1 inhibition restores T‐cell cytotoxicity suppressed by CAFs in ovarian cancer cells. (A) Flow cytometric analysis of CAOV3 ovarian cancer apoptosis under different coculture conditions. CAOV3 cells were cultured alone (top left), with OKT3‐activated PBMCs (15 ng·mL^−1^, 5 days) (top right), with activated PBMCs and CAFs (bottom left), or with activated PBMCs, CAFs, and epacadostat (500 nm, 5 days) (bottom right). Apoptosis was assessed by FITC‐Annexin V and PI staining. Early apoptotic (Annexin V+ PI−) and late apoptotic/necrotic (Annexin V+ PI+) cell percentages are shown in quadrants. (B) Quantified apoptotic CAOV3 cells under different treatment conditions. Bars indicate mean apoptotic cell percentages (Annexin V+) from experiments using three CAFs (OV01, OV02, and OV05). Statistical significance was determined using one‐way ANOVA followed by Tukey's multiple comparison tests (*P < 0.05, **P < 0.01, ***P < 0.001, ****P < 0.0001, and ns = not significant).

To further explore CAF immunosuppressive mechanisms, ovarian cancer apoptosis assays were conducted in T‐cell and CAF coculture experiments. CAOV3 ovarian cancer cells were grown for 5 days with activated PBMCs and CAFs in the presence/absence of epacadostat. CAOV3 cells showed elevated apoptosis levels when cocultured with activated T cells, whereas T‐cell‐mediated CAOV3 apoptosis was significantly reduced when cocultured with CAFs and activated T cells. Furthermore, epacadostat restored CAOV3 apoptosis levels that had been reduced by CAFs (Fig. 6A,B). These results suggested that epacadostat effectively overcame CAF‐mediated immunosuppression, leading to increased apoptosis.

## Discussion

We investigated CAF immunosuppressive functions in HGSOC and examined if IDO1 inhibition overcame this suppression in patients' tissue samples and cell lines. We observed that CAFs appeared to suppress T‐cell function, while IDO1 inhibition effectively reversed this immunosuppression.

CAFs isolated from patients with HGSOC were analyzed, after which known CAF markers were confirmed [10]. Real‐time PCR revealed higher mRNA levels of candidate markers (e.g., ACTA2, FAP, PDGFRA, PDGFRB, ITGA11, and TNC) in CAFs than in ovarian cancer cell lines, except for FSP1 and PDPN (Fig. 1A). Flow cytometry revealed strong α‐SMA (>89%) and minimal EpCAM (<2.3%) expression in all CAFs, confirming their fibroblastic and nonepithelial phenotype (Fig. 1B). Although healthy fibroblast controls were unavailable, the variability in CAF marker expression among samples aligns with the heterogeneity observed in single‐cell transcriptomic studies [35, 36].

This heterogeneity was recently identified by single‐cell RNA sequencing, which identified diverse CAF subpopulations in ovarian cancer, including myofibroblastic CAFs (myCAFs), inflammatory CAFs (iCAFs), and antigen‐presenting CAFs (apCAFs), each with unique functional characteristics [10, 36]. In particular, iCAFs were found to secrete inflammatory cytokines (e.g., interleukin‐6 (IL‐6)), thereby contributing to an immunosuppressive microenvironment [35, 36]. Although we did not subtype CAFs, the upregulation of IL‐6 and other immunosuppressive cytokines following T‐cell interaction suggests functional similarity to iCAFs. Future single‐cell analyses could clarify these subtype‐specific roles.

Increased immunosuppressive molecule (e.g., *IDO1*, *COX2*, and *PD‐L1*) levels in CAFs in coculture with activated T cells demonstrated the adaptive acquisition of immunosuppressive functions by these cells in the TME. In particular, IDO1 was shown to have key roles in immune suppression via tryptophan metabolism [37, 38]. Additionally, Zhao et al. [31], in their hepatocellular carcinoma (HCC) study, reported that CAFs induced IDO‐producing regulatory dendritic cells (DCs) via IL‐6‐mediated signal transducer and activator of transcription 3 (STAT3) activation. These DCs showed elevated IDO levels, which suppressed T‐cell proliferation and promoted regulatory T‐cell expansion. This mechanism enhanced the immunosuppressive environment in HCC, highlighting an IDO role in CAF‐mediated immune suppression.

We observed that inhibited CD4+ and CD8+ T‐cell proliferation and increased PD‐1 levels upon CAF coculture directly suppressed T‐cell function by CAFs, consistent with recent studies. Ford et al. [39] reported that CAF‐rich tumors excluded CD8+ T cells from the tumor stroma, resulting in poor anti‐PD‐1/PD‐L1 immunotherapy responses. Furthermore, Lakins et al. [40] demonstrated that CAFs can directly induce antigen‐specific deletion of activated CD8+ T cells through PD‐L2 and FASL‐mediated interactions, thereby promoting tumor cell survival and further supporting the immunosuppressive role of CAFs in the tumor microenvironment.

The ability of epacadostat to restore T‐cell proliferation and CD8+ T‐cell proportions suppressed by CAFs indicated a central role for IDO1 in CAF immunosuppression, consistent with recent studies showing the profound impact of IDO1 on the TME. For instance, an ovarian cancer study reported that elevated IDO1 levels correlated with lower Trp:Kyn ratios in the TME and also decreased CD8+ tumor‐infiltrating lymphocyte (TIL) infiltration [41]. Moreover, reduced PD‐1 levels in CD8+ T cells after epacadostat treatment suggested that IDO1 inhibition potentially influenced immune checkpoint molecule expression, indicating possible synergistic effects with immune checkpoint inhibitors. This observation aligned with head and neck cancer data where epacadostat significantly increased Natural Killer and CD4+ T‐cell migration and infiltration toward cancer cells [42]. This potential synergy between IDO1 inhibition and immune checkpoint blockade was further supported by a preclinical glioblastoma study where an IDO1 inhibitor combination with anti‐PD‐1 therapy and radiation led to durable survival benefits [43]. Thus, IDO1 appeared to be critical for modulating immune responses in the TME, while IDO1 inhibition was a possible strategy enhancing immunotherapy efficacy.

Reduced T‐cell Akt phosphorylation by CAFs and its restoration by IDO1 inhibition emphasized a key role for the IDO1–Akt axis in CAF‐mediated immunosuppression. While previous studies reported mechanistic links between IDO1 activity and PI3K/AKT signaling in cancer cells, thus promoting tumor growth and therapy resistance [44, 45], we extended these findings to T cells. We showed that IDO1's role in modulating tryptophan metabolism directly impacted T‐cell function via Akt signaling, affecting T‐cell exhaustion and regulatory T‐cell expansion. This novel insight into the IDO1–Akt axis in T cells provides a more detailed understanding of CAF‐induced immunosuppression, and underscores how targeting this axis in cancer and T cells could overcome immunosuppression and improve antitumor immunity.

These findings have important implications for ovarian cancer treatment. Currently, standard treatments for ovarian cancer primarily consist of cytoreductive surgery combined with chemotherapy [46]; however, targeted molecular therapies are rapidly evolving. In particular, Poly (ADP‐ribose) polymerase (PARP) inhibitors and anti‐angiogenic agents (e.g., bevacizumab) have shown efficacy for ovarian cancer treatment [47, 48, 49]. Our results suggest that targeting CAFs, particularly by inhibiting IDO1, could effectively enhance existing therapies [50].

However, this study had several limitations. The sample size was relatively small, and experiments were mainly performed *in vitro* without *in vivo* validation, which may limit the generalizability of our findings. Additionally, our patient cohort showed a high prevalence of BRCA1/2 mutations (75%), which exceeded the typical population rates. Genetic heterogeneity, including variations in BRCA and TP53 status, has not been systematically analyzed, further limiting its applicability across ovarian cancer subtypes. Therefore, caution is warranted when extrapolating the observed CAF‐mediated immunosuppressive mechanisms to all ovarian cancer subtypes and genetic backgrounds.

Future research should validate these findings using animal models, conduct analyses of CAF heterogeneity and subtype‐specific functions via single‐cell transcriptomic or proteomic approaches, and assess the efficacy of combining IDO1 inhibitors with immunotherapies in larger, histotype‐ and mutation‐stratified patient cohorts. Moreover, stratified analyses based on BRCA1/2 and TP53 mutation status are needed to determine whether CAF‐IDO1 interactions vary according to genetic background, thereby guiding the development of more precise and personalized therapeutic strategies.

In conclusion, CAFs derived from HGSOC exert potent immunosuppressive effects, primarily via an IDO1‐mediated mechanism. Treatment with the IDO1 inhibitor epacadostat effectively restored suppressed T‐cell proliferation and CD8+ T‐cell populations, modulated PD‐1 expression, and restored Akt signaling. These findings highlight the pivotal role of the CAF‐IDO1 axis in shaping the immunosuppressive tumor microenvironment and support targeting of IDO1 as a promising approach to enhance existing immunotherapeutic and standard treatment strategies for ovarian cancer.

## Conflict of interest

The authors declare no potential conflicts of interest.

## Author contributions

YJC conceptualized and supervised the research. HL and YJC designed the experiments. HL, JYH, and ISH conducted the experiments and analyzed the data. HL drafted the original manuscript. HL, JYH, ISH, and YJC reviewed and edited the manuscript. All authors read and approved the final manuscript.

## Supporting information


**Table S1.** List of antibodies used for flow cytometry.
**Table S2.** List of primer sequences used for real time PCR.

## Data Availability

The data supporting the findings of this study are provided within the manuscript and its Supporting Information. Further queries can be directed to the corresponding author.
